# Rasch Modelling to Assess Psychometric Validation of the Knowledge about Tuberculosis Questionnaire (KATUB-Q) for the General Population in Indonesia

**DOI:** 10.3390/ijerph192416753

**Published:** 2022-12-14

**Authors:** Ikhwan Yuda Kusuma, Deny Nugroho Triwibowo, Arik Dian Eka Pratiwi, Dian Ayu Eka Pitaloka

**Affiliations:** 1 Center of Excellence in Higher Education for Pharmaceutical Care Innovation, Universitas Padjadjaran, Sumedang 45363, Indonesia; 2 Pharmacy Study Program, Faculty of Health, Universitas Harapan Bangsa, Purwokerto 53182, Indonesia; 3 Institute of Clinical Pharmacy, Faculty of Pharmacy, University of Szeged, 6720 Szeged, Hungary; 4 Faculty of Pharmacy, STIFAR Yayasan Pharmasi Semarang, Semarang 50192, Indonesia; 5 Department of Pharmacology and Clinical Pharmacy, Faculty of Pharmacy, Universitas Padjadjaran, Sumedang 45363, Indonesia

**Keywords:** tuberculosis, knowledge, Rasch analysis, validation

## Abstract

Objective: This study aims to validate and evaluate the psychometric properties of the knowledge about tuberculosis questionnaire (KATUB-Q) for the general population in Indonesia. Methods: The KATUB-Q consists of three domains: general knowledge, transmission, and treatment, with 20 dichotomous items. Rasch analysis through WINSTEPS was used. Results: A total of 504 respondents from 34 provinces in Indonesia completed the survey. Based on the model fit statistics, 3 misfit items were deleted and 17 items were used. Item and person reliability, as well as Cronbach’s Alpha values were 0.99, 0.63, and 0.73, respectively, which means they achieved the minimum acceptable limit of 0.6. Based on the results, Indonesia’s Person ability analysis indicated a high level of knowledge. KATUB-Q has no significant bias item based on sex found in the differential item functioning analysis. Conclusion: KATUB-Q has 17 items with a valid and reliable instrument; hence, it can be used to measure the knowledge about TB in the general population. Practice implications: The unidimensional structure of the core items of the KATUB-Q provides empirical evidence for using the sum score of the items in practice to evaluate the effectiveness of TB education in the general population.

## 1. Introduction

Tuberculosis (TB), a bacterial infection caused by Mycobacterium tuberculosis, remains a major global health concern [1]. In 2021, an estimated 10 million people were infected with TB globally, with the most number of new cases occurring in the WHO South-East Asian Region [1]. Indonesia ranks third among countries with the highest incidence of TB, with approximately 824 thousand cases in 2020 [1]. Meanwhile, the transmission can be prevented and reduced by addressing the general population’s lack of knowledge [2]. Lack of knowledge about TB causes patients to underutilize health services, delay seeking diagnosis, and adhere poorly to treatment [3,4].

Several instruments, such as structured surveys, pre-validated questionnaires, and the quantitative survey Quality of Life Instruments for Chronic Diseases (QLICD-PT), have been created to assess public knowledge about TB [5,6]. Although TB knowledge questionnaires have been used for over a decade, rigorous analyses of their psychometric properties are lacking [5]. The majority of the available studies focused mainly on their internal consistency and test–retest reliability [5,6]. However, the structural validity of the TB knowledge test has not been assessed through psychometric analysis of the general population.

Some of the studies also focused primarily on the knowledge of health professionals, TB patients, and students [2,7,8]. The most popular method for evaluating structural validity is factor analysis [9]. However, the TB knowledge questionnaire produces binary response data which cannot satisfy the normal distribution assumption [9]. To assess structural validity using traditional factor analysis with binary response data, an alternative statistical method of factor analysis with a tetrachoric correlation matrix or less popular statistical software programs is required. These approaches and software programs have presumably limited the evaluation of structural validity for the TB knowledge questionnaire [8].

Rasch analysis has recently expanded into the health sciences as a modern psychometric method for investigating measurement properties such as structural validity, internal consistency, and measurement invariance in education [9]. It is based on the principle that the probability of an individual responding correctly to a binary response item is dependent on both the ability, including TB knowledge, and the item difficulty as measured by a logit scale [9]. Rasch analysis has been performed for psychometric evaluations to assess knowledge of multiple sclerosis, diabetes, nutrition, rheumatology, and human papillomavirus [7,8,9,10,11]. However, its ability for measuring TB knowledge is extremely uncommon. This study aims to assess knowledge about TB in Indonesian’s general community using Rasch analysis to demonstrate the validity of the questionnaire. Based on the literature review performed, this is the first study to perform Rasch analysis on a large population in Indonesia.

## 2. Methods

This study referred to the study by Zamanzadeh et al. [12] in designing the questionnaire and testing its validity as well as internal consistency. The instrument was developed in a two-step process, namely, development and judgment. In the first step, domain determination, sampling or item generation, and instrument formation were performed, while in the second step, face and content validity were tested.

### 2.1. Ethical Approval and Consent to Participate

The study was approved by the Health Research Ethics Committee of Universitas Harapan Bangsa (B.LPPM-UHB/956/05/2022) in May 2022, and written informed consent was obtained from all the participants. Anonymity of the participants was assured to protect their identities.

### 2.2. Item Development

The KATUB-Q instrument was developed through a three-step process: determining the content domain, sampling from content, and instrument formation. The first step aims to identify the content domain of the particular construct the instrument was designed to assess. The content domain refers to the subject area associated with the considered variables; it can be determined by reviewing the existing literature on the issue being assessed. This study adapted previous questionnaires [12,13], studies [14], and TB guidelines by WHO [15,16] to construct the framework and produce the questions.

The KATUB-Q instrument comprises three domains, namely, general knowledge, transmission, and treatment for tuberculosis. After developing a good understanding of the study’s theoretical framework problems through a literature review, items were created in each domain that can effectively answer the objectives. Each item in the questionnaire was developed based on the content domains and variables. The team members reviewed and approved the final preliminary version of the instruments. A total of 20 questions was further tested in the next stage for their face and content validity.

### 2.3. Face and Content Validity

The content validity of an instrument can be assessed using the expert panel’s judgments, this panel is comprised of content and lay experts. Potential study subjects are lay experts, while content experts are professionals or who work on the topic. Face validity was used to evaluate the acceptability of the statement constructs for each item’s phrasing, structure, orderliness, and scoring forms [17]. The experts assessed the questions individually and as a questionnaire tool, with respect to their content relevance and simplicity. They were requested to rate each item using two four-point ordinal Likert rating scales on relevance with the following; grade 1 = not relevant, 2 = somewhat relevant, 3 = quite relevant, and 4 = highly relevant, and clarity as follows 1 = not clear, 2 = need some revision, 3 = clear or need minor revision, and 4 = very clear. Furthermore, the scale was dichotomized into relevant ratings of 3 and 4 as well as those not relevant for 1 and 2. Item level content validity was determined as the number of experts giving a rating of 3 or 4 (agreed) divided by the total number of experts.

The content validity index (CVI) approach was used to measure experts’ feelings about the content validity of the scales [18]. It is the most frequently reported method for determining the content validity of an instrument and can be assessed using the Item-CVI (I-CVI) and the Scale-level-CVI (S-CVI). I-CVI was calculated by dividing the number of experts who rated each item as “highly relevant” by the total number of experts. When I-CVI is more than 0.79, the item is relevant, for values ranging from 0.70 and 0.79, the item requires revisions, while those below 0.70 can be omitted. Similarly, S-CVI was determined according to the number of items in a tool rated as “highly relevant.” It can be assessed by two methods, i.e., universal agreement (UA) among experts (S-CVI/UA), and the average CVI (S-CVI/Ave); the latter is a less conservative method. The difference between S-CVI/Ave and S-CVI/UA is determined by adding all items with I-CVI values of 1 and dividing by the total number of items, respectively. The S-CVI/UA 0.8 and the S-CVI/Ave 0.9 have excellent content validity [18], and specifically; for 6 experts, the acceptable CVI values are at least 0.83 [19].

### 2.4. Participants

This study used a quantitative approach with a cross-sectional survey design [14] with a total of 504 respondents from 34 provinces in Indonesia. Inclusion criteria in this study include Indonesian citizens aged 15–64 years, use and understand the Google form platform (Supplementary Materials), read well, and willing to fill out informed consent for approval as respondents. Meanwhile, the exclusion criteria were respondents who were not willing to fill out the questionnaire and those who did not completely provide information on the questionnaire form. The KATUB-Q is a self-administered instrument constructed by the Google forms platform. It was distributed through WhatsApp Messenger and Facebook Groups for three weeks. All participants were given 20 min to complete the questionnaires.

### 2.5. Psychometric Analysis

A psychometric analysis was developed based on the Rasch measurement model, which was analyzed using Winsteps software with a logit scale. The Joint Maximum Likelihood Estimation (JMLE) equation was used to execute the Winsteps Rasch analysis. This psychometric probability-based analysis method checks to confirm whether the observed responses match the pattern of the Rasch model [20]. Furthermore, Rasch analysis can determine the scale’s measurement and structural characteristics when the conditions of the model are satisfied. It is also applicable to determining the difficulty level of each item on the scale and the skills people need to measure certain things [19,20,21]. In the fields of health and education, Rasch modelling is frequently used to evaluate the psychometric qualities of scales, test items, and questionnaires [20,21]. The Rasch analysis properties were assessed by Winsteps software (defined in Appendix A).

## 3. Results

### 3.1. Face Validity and Content Validity

The face and content validity were used to verify that only relevant variables were selected and included in the structure of the draft instruments. Six experts comprising three pharmacists, two public health professionals, and one nurse provided feedback and contributions on the draft instrument, which were incorporated into the instrument’s development. All 20 items obtained content validity indexes (CVI) > 0.80 for relevance, clarity, simplicity, and comprehensiveness, as determined by the content validity approach [19]. Summary CVIs (S-CVIs) averaged between universal agreement (UA) and the average CVI (S-CVI/Ave) approaches were in the range of 0.85 and 0.98, respectively (Appendix B).

### 3.2. Participants

The participants in this preliminary study were 504 participants from 34 provinces in Indonesia (Appendix C). The samples were recruited by using convenience sampling. In this study, there were participants from each province for the analysis. Among the participants who completed the questionnaire, 72.0% were females; 62.9% were 17–25 years old; 95.8% had no history of TB; and 53.6% live in rural populations. The KATUB-Q analysis samples were from 34 provinces in Indonesia. Although the sex demographic population is unbalanced in this study, the mean logit scores of female and male participants are not significantly different. The female group has 1.55 logits, and the male group has 1.20 logits. The difference is less than 1 logits. Therefore, we assumed that it is still comparable to measure in this study. However, for specific item investigation, we present the DIF analysis in Section 3.8.

### 3.3. Psychometric Analysis

The psychometric properties of the developed instrument based on the Rasch measurement model were analyzed. Winsteps runs analyses was based on Joint Maximum Likelihood Estimation (JMLE) equations. In this formulation, the raw data were converted to interval data (logit) [22,23]. The logit scale can express a person’s ability and item difficulty ranging from positive to negative infinity. The 20-item KATUB-Q and 504 participants from Indonesia were analyzed as two-facet items and person models using the Rasch measurement with the Winsteps software [24]; fit indices for the items included in the model as presented in Table 1.

### 3.4. Dimensionality

The observed raw variance explained by the measure was 40.8%, while the unexplained variance was 14.0%. The instrument can reach unidimensionality when the measure explains more than 30% of the raw variance [23,25]. Based on this evidence, it was concluded that the assumption of one-dimensionality was met and the unexplained variance confirmed unidimensionality, which is a fundamental requirement for the Rasch model.

### 3.5. Item and Person Correlation

**Figure 1:**
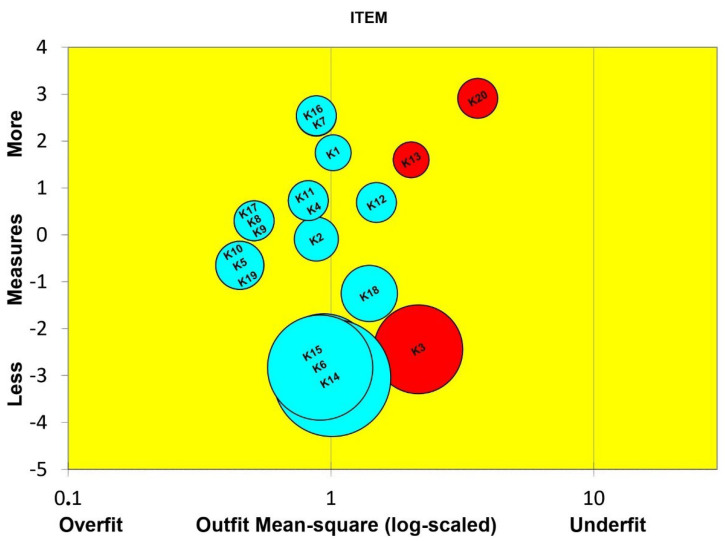
Bubble chart of item fit order based on Infit MNSQ; Y axis is JMLE measure; X axis is item fit mean square; overfit (x >1.50); outfit (0.50 < x < 1.50). Each bubble represents an item whose size is proportional to the standard error of item difficulty calibration. Well-fitting items are close to the central vertical line.

Overall, the means of infit (weight) and outfit (unweight) mean square (MNSQ) for the respondents were acceptable, with values of 0.86 and 0.61, respectively, while those of z-standard (ZSTD) were also acceptable, with values of −0.41 and −0.16, respectively. Meanwhile, infit and outfit MNSQ for items were 0.90 and 0.82, respectively, while those of infit and outfit ZSTD were −2.14 and −2.10, respectively. The analysis found several misfitting items with the value of MNSQ above the threshold which ranges from 0.5 to 1.5, i.e., K20 (3.6224), K3 (2.1478), and K13 (2.0213) (Table 2) and were removed. Afterward, all 17 items were found to have a good fit for the model (Figure 1). There were also some item/person misfits based on the ZSTD threshold (n = 20) but they can be ignored because the samples were more than 200 [26].

### 3.6. Reliability and Internal Consistency

**Figure 2:**
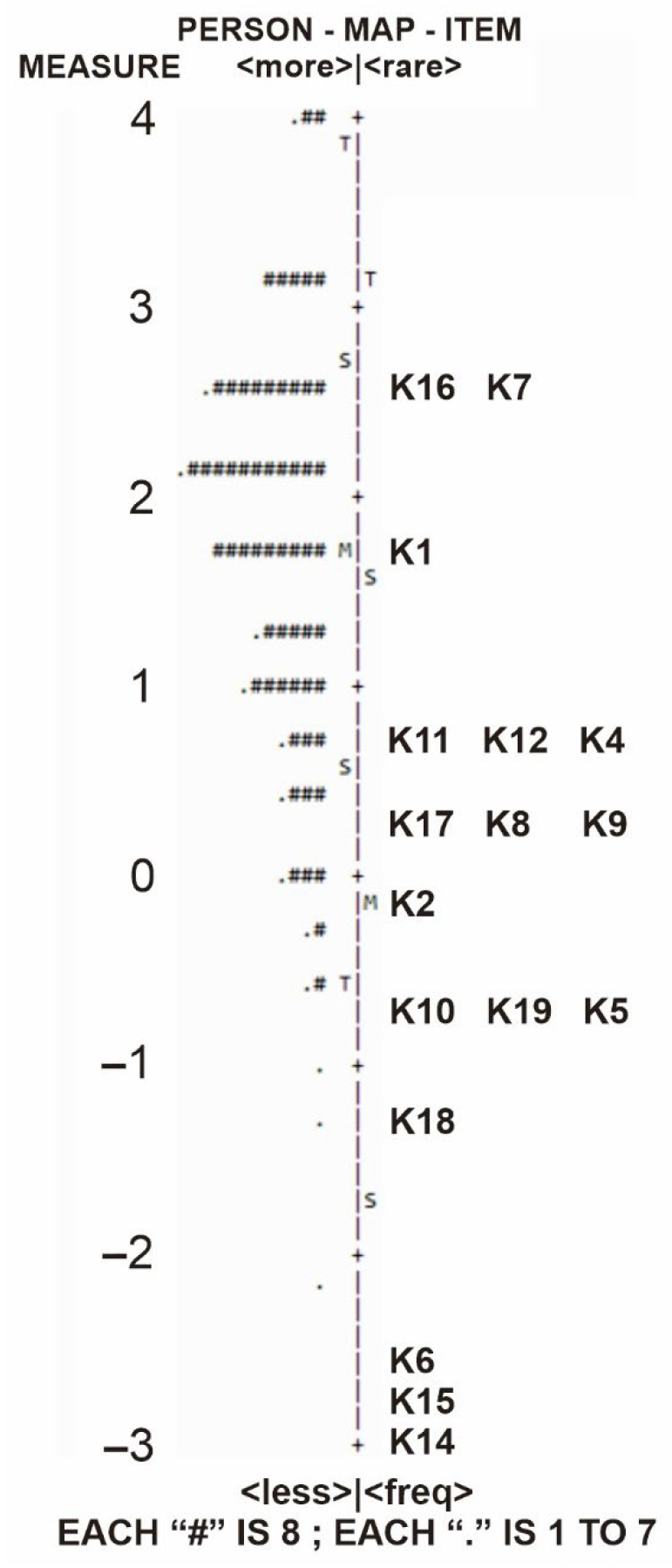
Wright map (item–person correlation). The right-hand side shows the 17 items of the questionnaire, from the easiest (K14, bottom indicator) to the most difficult items (K16 and K7, top indicator); the left-hand side locates the participant’s knowledge level measured along with the items. The Ms on the right and left-hand sides indicate the mean item and the mean person measures, respectively. The closer the location of M is to both measures, the better [31]. “#” represents 8 items and “.” represents 1–7 participants.

Person and item reliability on the KATUB-Q were reported as satisfactory, the person separation index was 1.31, while the person reliability index was 0.63. This indicates that there were enough participants to distinguish between those with higher and lower abilities. The index of item separation was 10.98, and the item reliability was 0.99 [27]. This shows that the sample size was sufficient to demonstrate the continuum of item difficulty shown in Figure 2. Furthermore, the internal consistency of the final 17 questions on the KATUB-Q was acceptable based on Cronbach’s alpha = 0.73 [28], showing that they consistently measure the unidimensional construct, i.e., knowledge of TB.

### 3.7. Person Ability Distribution

**Figure 3:**
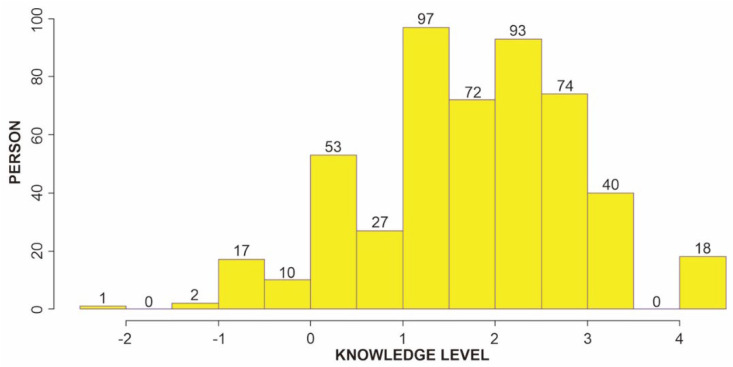
Histogram chart of person knowledge ability about tuberculosis.

Overall, the percentage of the observed count can be found in the output section of Winstep software in the summary of a dichotomous rating scale, or partial credit structures that explain the count of correct or incorrect answers from the total of all categories. Based on the results, most of the participants had good knowledge of TB. In this case, the more positive logit value indicates a high level of knowledge. The results show that respondents prefer the positive logit with an estimated 474 people in total to the negative logit with 110. The positive logit consisted of 80, 169, 167, and 48 people with values of 0–1, 1–2, 2–4, and >3, respectively, while 27 people had a logit value of 0–(−1), and 3 people had a value lower than −1 (Figure 3).

### 3.8. Differential Item Functioning (DIF) Analysis

**Figure 4:**
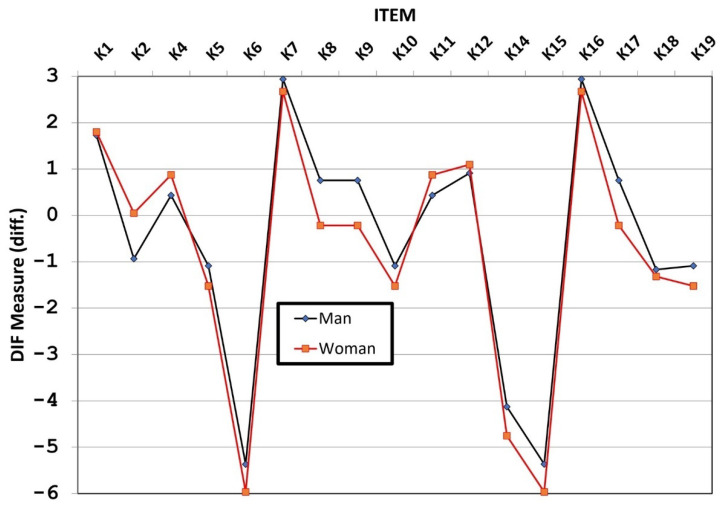
Differential item functioning based on sex.

DIF analysis was conducted to check for the possibility of item bias based on sex; a good Rasch model should not systematically differ (i.e., DIF) based on relevant personal characteristics [29]. The results show that the items K2 (−0.98), K8 (0.97), K9 (0.97), and K17 (−0.97) have DIF bias with moderate to large category (|DIF| ≥ 0.64 logits) and prob (|DIF| ≤ 0.43 logits) ≤ 0.05 or 2-sided (Figure 4). Items with bigger values for the DIF measure indicate higher difficulty for the group [27]. Item K8, K9, and K17 are more difficult for males as indicated by the bigger values of the DIF measure when compared to females, and only item K2 was more difficult for the females than males. However, these items can be retained because they are still reliable and valid, and it was not eliminated because this action could lead to lowering the reliability and validity [30].

## 4. Discussion

This study used the dichotomous model of Rasch analysis to examine the psychometric properties of structural validity, internal consistency, and measurement invariance for the following core items of the KATUB-Q: general knowledge, transmission, and treatment for TB. Based on the results, all items except for a single misfitting item exhibited a unidimensional structure. Considering that a total score is only appropriate when a construct to be measured is unidimensional or a subscale [31], the structural validity result provides an empirical rationale for summing all the scores for the core items of KATUB-Q.

The person–item map visually displays how the distribution of item difficulty is matched to the ability of a person [20]. Based on the results, the distribution width for the abilities of persons was not well matched with that of the item difficulties. Specifically, there were no items aligned with about half of the people exhibiting the widest range of abilities, implying that the instrument does not contain sufficiently difficult items. Therefore, it is recommended that items of greater difficulty be added to the present 17 items of KATUB-Q.

The well-known method used to assess internal consistency is Cronbach’s alpha in classical test theory. The development study of the KATUB-Q found that Cronbach’s alpha was 0.73 for the core items measuring the construct of general TB knowledge. Meanwhile, the values found in populations of Malaysian, Turkish, and Korean patients were 0.70, 0.60, and 0.67, respectively [13,14,30], which did not satisfy the criterion of >0.70 for internal consistency.

Person reliability is less well known than Cronbach’s alpha, and it corresponds to the internal consistency within the modern test theory of Rasch analysis, which can be interpreted similarly to the traditional assessments. In other words, the person reliability is known to be a conservative and better assessment of internal consistency than the Cronbach’s alpha [32]. The item reliability and separation index were satisfied, inferring that general TB knowledge exhibits a reproducible item-difficulty hierarchy.

DIF indicates whether items are used in the same way across groups [33]. Based on the results, four items have moderate to large DIF bias, namely, K2: fever and cough for more than two weeks is a symptom of TB, K8: BCG vaccine can be used for TB prevention, K9: smoking increases the risk of TB, and K17: TB treatment is carried out for 1–2 weeks. The assessment of knowledge about TB conducted in a previous study showed that a sizeable number lacked the understanding of ensuring optimal TB medication adherence to prevent further transmission [34]. Another study revealed that about 75% and 2% of respondents indicated that they visited faith and traditional healers respectively after diagnosis [35]. These results mean that careful interpretation is required when using the four items in practice. Moreover, it demonstrates the need for health education efforts to strengthen accurate information which can improve knowledge and correct misconceptions about TB among patients within the community.

### Practice Implications

We provide a validated, reliable tool for measuring knowledge in the Knowledge About Tuberculosis Questionnaire (KATUB-Q) for the general population to identify deficits in TB knowledge and evaluate TB knowledge change in the community. These statements result from Rasch parameters whereby the unidimensional structure of the core items of the KATUB-Q provides empirical evidence for using the sum score of the items in practice. For example, the total score can be used to evaluate the effectiveness of TB education in the general population. This study applied Rasch analysis to the KATUB-Q to assess its structural validity, internal consistency, and measurement invariance. Therefore, further applied research should focus on TB’s responsiveness, which is the ability to detect changes over time and can be assessed using a longitudinal study design.

## 5. Conclusions

This study used Rasch analysis to evaluate the psychometric properties of the core-item KATUB-Q in the general population of Indonesia. It was performed to assess knowledge about tuberculosis in general terms, transmission, and treatment. Overall, the means of infit (weight) and outfit (unweight) of the mean square and z-standard for the respondents and items were acceptable. From 20 items, the analysis found three misfit items, and 17 items were found to have a unidimensional structure and contributed to how suitable the KATUB-Q was for measuring the underlying construct of general TB knowledge. The core-item KATUB-Q demonstrated item and person reliability, as well as Cronbach’s alpha values, which achieved the minimum acceptable limit, and indicated a high level of knowledge. In addition, KATUB-Q has no significant bias item based on sex found in the differential item functioning analysis. These findings indicate that this instrument can be used to measure the knowledge about TB in the general population.

## Figures and Tables

**Table 1:** The summary statistics of Rasch parameters for KATUB-Q.

Criteria	Person	Item (Question)
N	504	17
Mean:		
Infit MNSQ	0.86	0.90
Infit ZSTD	−0.41	−2.14
Outfit MNSQ	0.61	0.82
Outfit ZSTD	−0.16	−2.10
Reliability (Rasch)	0.63	0.99
Reliability (Cronbach’s alpha)	0.73	
Separation coefficient	1.31	10.98
Unidimensionality		
Raw variance by measure	40.8%	
Unexplained variance in 1st contrast	4.02%	

MNSQ = mean square, ZSTD = z-standard.

**Table 2:** Item fit statistics for KATUB-Q items from the dichotomous Rasch analysis (n = 504).

Item	Description	Infit		Outfit		Point Measure Correlation
		MNSQ	ZSTD	MNSQ	ZSTD	
K20	TB drugs consist of more than 1 type of drug	1.5493	9.2615	3.6244	9.9036	−0.1866
K3	Fever and cough more than 2 weeks is a symptom of TB	1.0746	0.4611	2.1478	2.6021	0.0896
K13	TB can be spread by touching goods in public facilities	1.6427	9.9016	2.0213	9.902	−0.0375
K12	Weather changes increase the risk of transmission	1.4519	8.8815	1.493	6.1715	0.1538
K18	Discontinued the TB drugs can increase the risk resistance and severity	1.1306	1.3511	1.3986	1.7814	0.209
K1	TB caused by *Mycobacterium tuberculosis*	1.0035	0.111	1.0158	0.291	0.4646
K14	Cough from a TB patient without covering the mouth can increase the risk of transmission	0.9926	0.061	1.0071	0.161	0.1447
K6	TB can attack organs other than the lungs	1.0486	0.291	0.9404	−0.0191	0.1384
K15	TB can be treated	1.0428	0.261	0.9143	−0.0891	0.14
K7	Infection can happen again to someone who has had TB	0.8972	−2.4591	0.8845	−1.3591	0.4992
K2	TB caused by a viral infection	0.9441	−0.9791	0.8828	−1.0891	0.475
K16	Use of herbal medicines with TB drugs can improve outcome	0.8942	−2.5291	0.8823	−1.3791	0.5007
K4	TB is a hereditary disease	0.909	−2.0991	0.8227	−2.7292	0.5492
K11	TB can be transmitted through breast milk	0.9089	−2.0991	0.8223	−2.7392	0.5498
K8	BCG vaccine used for TB prevention	0.6416	−8.2094	0.5066	−6.9695	0.7176
K9	Smoking increases the risk of TB	0.6416	−8.2094	0.5066	−6.9695	0.7176
K17	TB treatment is carried out for 1–2 weeks	0.6413	−8.2194	0.5064	−6.9795	0.7181
K19	TB treatment can cure by resting without taking drugs	0.7316	−4.1593	0.4546	−4.5795	0.5916
K5	TB can be diagnosed by a sputum test	0.7315	−4.1593	0.4545	−4.5795	0.5913
K10	Shaking hands with a TB patient increase the risk of infection	0.7315	−4.1593	0.4545	−4.5795	0.5913

## Supplementary Materials

The following supporting information can be downloaded at: https://www.mdpi.com/article/10.3390/ijerph192416753/s1, Knowledge about Tuberculosis Questionnaire (KATUB-Q) for the General Population in Indonesia.

## Appendix A. Summary Statistics of Rasch parameters for KATUB-Q

**Table:**

**Rasch Measurement Threshold**	**Definition**	**Statistical Test in Winsteps**
Dimensionality	The unidimensionality shows that the instrument measures the same dimension, which is student misconception in science	The value of the raw variance explained by the measure is more than 30%. Principal component analysis (PCA) of the residuals with first contrast eigenvalue of ≤2.0 indicates unidimensionality [25].
Fit Diagnosis	A test to indicate how accurately or predictably data fit the model, for both items and persons, and the whole subscale	The acceptable range for infit and outfit Mean Square (MNSQ) is from 0.5 to 1.5, where the ideal values for fit criteria are close to 1.00 logits [24,26], and the acceptable range for infit and outfit z-standardized (ZSTD) should range from −2 logits to 2 logits [24,27]. The acceptable reliability criteria range from 0.68 to 1.00 [24,27].
Reliability	Reliability (separation index) means “reproducibility of relative measure location” for person and items	The acceptable item/person reliability criteria range from 0.68 to 1.00 [24,27], and person or item separation index of ≥1.5 indicates good internal consistency and reliability. (Linacre, 2019). The acceptable values of Cronbach’s alpha are 0.7 or 0.6 [28].
Item-Person Correlation	A measure of the ability of the person to answer the KATUB items correctly against the difficulty level of the KATUB items arrayed along the same continuum using the same linear scale (logit scale).	Item-person interaction is displayed in the Wright map and Bubble chart. The right-hand side shows the items of the questionnaire from the easiest (bottom indicator) to the most difficult one (top indicator); the left-hand side locates the person’s ability measured along with the items [29]. Bubble chart illustrated infit MNSQ, Each bubble represents an item, whose size is proportional to the standard error of item difficulty calibration. Well-fitting items are close to the central vertical line [30].
Differential item functioning	Conducted to check whether there were items bias based on gender.	DIF analysis is divided into three types namely negligible (|DIF| 0–0.43 logits), slight to moderate (|DIF| ≥ 0.43 logits) and prob(|DIF| = 0 logits) ≤ 0.05 (2-sided), moderate to large (|DIF| ≥ 0.64 logits) and prob(|DIF| ≤ 0.43 logits) ≤ 0.05 (2-sided) [29].

Infit: inlier-sensitive fit; Outfit: outlier-sensitive fit; MNSQ: mean square; ZSTD: z-standard scores; DIF: differential item functioning.

## Appendix B. Face validity and content validity of the 20-items KATUB-Q

**Description**	**Panel 1**		**Panel 2**		**Panel 3**		**Panel 4**		**Panel 5**		**Panel 6**		**Expert in Agreements**	**I-CVI Score**	**S-CVI Score**
	**n**	**Code**	**n**	**Code**	**n**	**Code**	**n**	**Code**	**n**	**Code**	**n**	**Code**			
Item K1	4	1	4	1	4	1	4	1	4	1	4	1	6	1.00	1
Item K2	4	1	4	1	4	1	3	1	4	1	4	1	6	1.00	1
Item K3	4	1	2	0	4	1	3	1	3	1	4	1	5	0.83	0
Item K4	4	1	4	1	4	1	4	1	3	1	4	1	6	1.00	1
Item K5	4	1	3	1	4	1	4	1	3	1	4	1	6	1.00	1
Item K6	4	1	4	1	4	1	4	1	4	1	4	1	6	1.00	1
Item K7	4	1	4	1	4	1	4	1	4	1	4	1	6	1.00	1
Item K8	4	1	4	1	4	1	4	1	3	1	4	1	6	1.00	1
Item K9	3	1	4	1	4	1	3	1	4	1	4	1	6	1.00	1
Item K10	4	1	4	1	4	1	4	1	4	1	3	1	6	1.00	1
Item K11	4	1	4	1	4	1	3	1	4	1	4	1	6	1.00	1
Item K12	4	1	4	1	4	1	4	1	3	1	4	1	6	1.00	1
Item K13	3	1	3	1	2	0	4	1	4	1	4	1	5	0.83	0
Item K14	4	1	4	1	4	1	3	1	4	1	4	1	6	1.00	1
Item K15	4	1	4	1	4	1	3	1	4	1	4	1	6	1.00	1
Item K16	4	1	4	1	4	1	4	1	4	1	4	1	6	1.00	1
Item K17	4	1	4	1	4	1	4	1	4	1	4	1	6	1.00	1
Item K18	4	1	4	1	4	1	3	1	4	1	4	1	6	1.00	1
Item K19	4	1	4	1	4	1	4	1	4	1	4	1	6	1.00	1
Item K20	2	0	4	1	4	1	4	1	4	1	4	1	5	0.83	0
Mean		0.95		0.95		0.95		1.00		1.00		1.00	Total Agreement	19.50	17.00
													S-CVI/Ave	0.98	
													S-CVI/UA		0.85

## Appendix C. Sociodemographic of KATUB-Q Study Participants

**Category**		**Number (N)**	**Percent (%)**
Age	17–25 years	317	62.9
	26–35 years	118	23.4
	36–45 years	49	9.7
	46–55 years	17	3.4
	56–65 years	2	0.4
	>65 years	1	0.2
Sex	Man	141	28.0
	Woman	363	72.0
TB history	Have history of TB	21	4.2
	Never	483	95.8
Residence	Rural	270	53.6
	Urban	234	46.4
