# Supplementary material for: Rasch Modelling to Assess Psychometric Validation of the Knowledge about Tuberculosis Questionnaire (KATUB-Q) for the General Population in Indonesia
Source: Int J Environ Res Public Health. 2022 Dec 14;19(24):16753. doi: 10.3390/ijerph192416753 (PMC9779046; doi:10.3390/ijerph192416753)
Supplement: Supplementary file 1 [file ijerph-19-16753-s001.zip › ijerph-1983932-supplementary.pdf]

Knowledge About Tuberculosis Questionnaire (KATUB-Q) For The General Population in Indonesia

You are invited to participate in a research study about Knowledge About Tuberculosis (TB) Questionnaire (KATUB-Q) for the general population in Indonesia. The aims of this research study is to develop questionnaire toward tuberculosis in Indonesian community. This study is being conducted by apt.Ikhwan Yuda Kusuma,M.Si. (University of Harapan Bangsa/University of Szeged), Deny Nugroho Triwibowo. M.Kom. (University of Harapan Bangsa), apt. Arik Dian Eka Pratiwi,S.Farm.,M.Si (STIFAR Semarang), and Dr. apt. Dian Ayu Eka Pitaloka,S.Farm.,M.Si. (Padjadjaran University) has provided funding for this study.

After get explanation from the researcher about this study. I invited to participate in a research project about Knowledge About Tuberculosis Questionnaire (KATUB-Q) for the general population in Indonesia. This online survey should take about 15 minutes to complete. I volunteer to take part in this research questionnaire, and responses will be kept anonymous to the degree permitted by the technology being used. I understand this research was approved by the Health Research Ethics Committee of Universitas Harapan Bangsa (B.LPPM-UHB/956/05/2022) in May 2022. Questions, concerns or complaints about this project or bene ts or risks associated with researcher this study can be answered by contacted to:

Name: apt. Ikhwan Yuda Kusuma, S. Farm. M.Si.

Contact Person:
+36 20 413 4166

---

* Required

1. I have read and I understand the provided information and have had the opportunity to ask questions. I understand that my participation is voluntary and that I am free to withdraw at any time, without giving a reason and without cost. I understand that I will be given a copy of this consent form. I voluntarily agree to take part in this study. *

   Mark only one oval.

   ◯ I'm Agree

PART ONE: DEMOGRAPHIC DATA

Please fill in based on your biography

2. Full Name (Example : *Cecep Darwis Mutaqqin*) *

3. Age *

Mark only one oval.

- 12 - 16 years old
- 17 - 25 years old
- 26 - 35 years old
- 36 - 45 years old
- 46 - 55 years old
- 56 - 65 years old
- > 65 years old

4. Gender *

Mark only one oval.

- Male
- Female

5. Phone Number *

6. Have you ever had Tuberculosis (TB)? *

Mark only one oval.

- Yes, I have
- Never

7. Province (Your Current Residence) *

Mark only one oval.

- Aceh
- North Sumatera
- West Sumatera
- Riau
- Jambi
- Riau Islands
- Bengkulu
- South Sumatera
- Bangka Belitung Islands
- Lampung
- Banten
- Jakarta
- Central Java
- West Java
- Yogyakarta
- East Java
- Bali
- West Nusa Tenggara
- East Nusa Tenggara
- West Kalimantan
- East Kalimantan
- Central Kalimantan
- South Kalimantan
- North Kalimantan
- South Sulawesi
- West Sulawesi
- North Sulawesi
- Southeast Sulawesi
- Central Sulawesi
- Gorontalo
- Maluku

North Maluku
Papua
West Papua

PART TWO: GENERAL KNOWLEDGE ABOUT TB

Please answer with TRUE or FALSE answer according to your opinion and knowledge on each of the questions below:

8. 1. TB caused by *Mycobacterium tuberculosis* *

Mark only one oval.

True
False

9. 2. TB caused by a viral infection *

Mark only one oval.

True
False

10. 3. Fever and cough more than 2 weeks is a symptom of TB *

Mark only one oval.

True
False

11. 4. TB is a hereditary disease *

Mark only one oval.

True
False

12. 5. TB can diagnosed by a sputum test *

Mark only one oval.

- True
- False

13. 6. TB can attack organs other than the lungs *

Mark only one oval.

- True
- False

14. 7. Infection can happen again to someone who have had TB *

Mark only one oval.

- True
- False

15. 8. BCG (Bacillus Calmette-Guérin) vaccine used for TB prevention *

Mark only one oval.

- True
- False

16. 9. Smoking increases the risk of TB *

Mark only one oval.

- True
- False

17. 10. Shaking hands with a TB patient increase the risk of infection *

Mark only one oval.

- False
- True

18. 11. TB can be transmitted through breast milk *

Mark only one oval.

- True
- False

19. 12. Weather changes increase the risk of transmission *

Mark only one oval.

- True
- False

20. 13. TB can be spread by touching goods in public facilities *

Mark only one oval.

- True
- False

21. 14. Cough from a TB patient without covering the mouth can increase the risk of transmission *

Mark only one oval.

- True
- False

22. 15. TB can be treated *

Mark only one oval.

- True
- False

23. 16. Use of herbal medicines with TB drugs can improve outcome *

Mark only one oval.

- True
- False

24. 17. TB treatment is carried out for 1-2 weeks *

Mark only one oval.

- True
- False

25. 18. Discontinued the TB drugs can increase the risk resistance and severity *

Mark only one oval.

- True
- False

26. 19. TB treatment can cure by resting without taking drugs *

Mark only one oval.

- True
- False

27. 20. TB drugs consist of more than 1 type of drug *

Mark only one oval.

- ( ) True
- ( ) False

PART THREE: FINAL COMMENT

28. Please inform us of any additional information, suggestions, or opinions you may be willing to share with us on the topic of this questionnaire (optional):

This content is neither created nor endorsed by Google.

Google Forms
